# Prevalence and Factors Associated with Early Sexual Initiation among College Students in Southwest Ethiopia

**DOI:** 10.1155/2020/8855276

**Published:** 2020-11-25

**Authors:** Tewodros Yosef, Tadesse Nigussie, Dawit Getachew, Melkamsew Tesfaye

**Affiliations:** Department of Public Health, College of Medicine and Health Sciences, Mizan-Tepi University, Mizan-Teferi, Ethiopia

## Abstract

**Background:**

Early age at first sexual practice is a public health issue and now common around the world especially in the developing countries. The development of effective strategies to reduce the adverse consequences of early sexual initiation becomes real when sufficient data is available. Therefore, this study is aimed at assessing the prevalence and factors associated to early sexual initiation among college students in southwest Ethiopia.

**Methods:**

A cross-sectional study was conducted among 453 college students in southwest Ethiopia from April to May 2018. A two-stage stratified sampling technique was used to select the study participants. The data were collected using structured pretested self-administered questionnaire. The collected data were entered using Epi-Data version 4.2.0.0 and analyzed using SPSS version 20. Logistic regression models were fitted to assess the effect of independent variables on the outcome variable. Significance was declared at *p* < 0.05 in the multivariable logistic regression analysis.

**Results:**

The proportion of early sexual initiation among college students was 17.9%, 95% CI (14.4%-24.4%). The mean age of sexual intercourse was 17.6 (±2 SD) years. Nearly three-fourths (73.4%) of the respondent's reason for early sexual intercourse was falling in love. More than half (62.2%) of the respondents used a condom for their first sexual intercourse. The factors associated with early sexual initiation were being female (AOR = 2.09and 95% CI [1.17-2.35]), chewing khat (AOR = 7.05 and 95% CI [3.81-13.1]), exposed to pornographic materials at age < 18 years (AOR = 3.57 and 95% CI [1.94-6.89]), and poor knowledge of sexually transmitted diseases (AOR = 8.69 and 95% CI [3.52-21.5]).

**Conclusion:**

The prevalence of early sexual initiation among college students was alarmingly high. This may be associated with a huge burden of poor sexual and reproductive health. Therefore, creating awareness of the factors and related negative sexual and reproductive health effect of early sexual initiation for young peoples through the use of mass media (television and radio), school teachers, and parents plays a paramount importance.

## 1. Introduction

Young adults are marked by a decreasing dependence on the family, growing importance of social relationships, and an intense period of experimentation and new experiences, which often include the onset of sexual activity [1, 2]. Sexual initiation usually happens during adolescence and early onset may result in unwanted consequences in several aspects. The unwanted consequences are due to the presence of factors associated between the early first sexual intercourse and the establishment of behavior patterns that may last for the entire life [3]. Youths begin their sexual activity while still attending elementary school [4].

Early sexual initiation is defined as an experience of first intercourse before 18 years of age [5–7]. Early age at first sexual practice is a public health issue [8] and now common around the world especially in the developing countries [9, 10]. Despite the age at sexual debut varies from place to place and among different individuals, however, early adolescent sexual activity remains as a problem with negative psychosocial and health outcomes [11, 12].

Early initiation of sexual activity affects the sexual and reproductive health of the young population [5, 13]. Youths who begin early sexual activity are more likely to have high-risk sex (multiple sexual partners) [8], and they are less likely to use condoms [14] which are related to the increased susceptibility to human immunodeficiency virus (HIV) and other sexually transmitted diseases (STDs) transmission, unwanted pregnancy, and its complications [5, 6, 9, 11, 13, 15–19]. Worldwide, the high prevalence of teenage pregnancies among in-school adolescent girls in particular is a major concern for majority of parents, guardians, and duty bearers [20–22]. In addition to sexual-related outcomes, early sexual intercourse is also associated with other unhealthy behaviors including alcohol use, delinquency, and violence [17, 23].

Worldwide, several studies revealed that gender [1, 6, 9–11, 24, 25], residence [7, 15, 26], religion [5, 7, 15, 20, 26–28], self and parental education [7, 24, 29], socioeconomic status [7, 20, 24, 29], drinking alcohol [6, 7, 9, 11, 24, 26, 28], chewing khat [5–7], cigarette smoking [25], watching pornographic materials at age < 18 years [5, 6, 9], visiting night or day party [9], classmate friend/peer pressure [5, 11, 15, 24, 30], being less connected with parents/defective parental monitoring and bonding [5, 6, 20, 25, 27, 30, 31], positive attitudes regarding condom efficacy [26] knowledge on family planning [7], and more positive attitudes to family planning use [26] were the factors associated with early sexual initiation.

Despite some sherds of evidence available in northern part of Ethiopia [5, 13, 25, 32], however, there is no study that shows the prevalence and associated factors of early sexual initiation among young adults in southwest Ethiopia. Besides, a previous study done in the study area showed that 51.3% of adolescent and young adults had risky sexual behavior [33], which happened before the age of 18 years. The avoidance of unwanted consequences of early sexual initiation can be done following the understanding of factors that influence adolescent early sexual decision-making [16]. The development of effective strategies to reduce the adverse consequences of early sexual initiation becomes real when sufficient data is available on the issue. Therefore, this study is aimed at assessing the prevalence and factors associated to early sexual initiation among college students in southwest Ethiopia.

## 2. Methods

### 2.1. Study Design, Setting, and Period

A cross-sectional study was conducted among college students from April 01 to 30, 2018. The college is found at 585 km southwest of Addis Ababa, the capital city of Ethiopia (the name and region of the college are being withheld to ensure the anonymity of the study subjects). The college was established in 2005. The college teaches students in ten departments, with five/four levels for each department. The college had a total of 1810 students (920 male and 890 female) during the study period [34].

### 2.2. Populations

The source population was all regular college students, who were attended their class during the study period. The study population was randomly selected students who studied during the study period.

### 2.3. Sample Size Determination and Sampling Techniques

The sample size was determined using a single population proportion formula, with an assumption of expected proportion of early sexual initiation to be 18.4% [5], 5% margin of error, 95% confidence interval, 10% for nonresponse compensation, and a design effect of 2. The final computed sample size was 486. A two-stage stratified sampling technique was used to select 486 regular students. In college, there were ten departments with five/four levels for each department. The departments were stratified based on levels (levels I-V). For each level, the sample size was proportionally allocated. Finally, the potential participants were selected using systematic random sampling.

### 2.4. Study Variables

The dependent variable was early sexual initiation. The independent variables were sex, residence, parental education, religion, family income, substance use (alcohol, cigarette, and khat), exposure to pornographic materials at age < 18 years, and knowledge of STIs.

### 2.5. Operational Definitions

- Age at sexual initiation is age at first intercourse (vaginal penile penetration), but other nonintercourse sexual contacts (kissing, dating, and men sex with men) were not included [5, 6, 25]
- Early sexual initiation was defined as an experience of first intercourse before 18 years of age [5–7]
- Sexually active was defined as a respondent who claimed to have engaged in sexual activity at least once prior to the study [5]
- Pornographic materials refer to newspapers, magazines, books, photographs, movies, and the internet intended to sexually arouse the viewer [5]
- Multiple sexual partners were defined as the behavior of a person with two or more sexual partners [34]

### 2.6. Data Collection Instrument and Procedures

The data were collected using structured pretested self-administered questionnaire. The questionnaire was developed by reviewing relevant literature. First, it was prepared in English, then translated it into the local language, and retranslated back it into English to check the consistency by an independent translator. A pretest was done on 5% of the total sample size in similar setups before the actual data collection was commenced. The training was given to data collectors and supervisors concerning the objective and process of data collection and to discuss the presence of an ambiguous question in the questionnaire.

### 2.7. Data Processing and Analysis

The collected data were entered into Epi-Data version 4.2.0.0 and analyzed using the SPSS version 20 statistical software. The final results were presented in tables and numerical summery measures (mean and standard deviation). Despite 180 respondents ever had sex, the prevalence of early sexual initiation was done from the whole study population. Of the 453 respondents, 180 had ever sex before. Because all study participants aged 18 years and above, those who had no sex before will have had sex in the future. Due to this, those who had no sex before were treated as late initiator (since all respondents aged 18 years and above). A binary logistic regression analysis was used to look for the association between outcome and independent variables. Variables with a *p* value of less than 0.25 in the bivariate logistic regression were included in the multivariable logistic regression. Finally, variables in the multivariable logistic regression with a *p* value <0.05 were considered as significantly associated with the outcome variable.

### 2.8. Ethical Consideration

Ethical clearance was obtained before conducting this research from the College of Medicine and Health Sciences, Mizan-Tepi University-Institutional Review Board (MTU-IRB). The study participants were informed about the purpose of the study, their right to deny participation, anonymity, and confidentiality of the information. The confidentiality of the response was maintained. Personal privacy and cultural norms were respected properly. Written informed consent was obtained from participants who participated in the study.

## 3. Results

### 3.1. Sociodemographic and Behavioral Factors

Out of 486, 453 students were interviewed yielding a response rate of 93.2%. Two hundred ten (46.4%) of respondents were male. Forty-six (10.2%) and 392 (86.5%) respondents were married and Christian in religious, respectively. One hundred ninety-two (42.4%) of the respondents were from rural. The mean age of the respondents was 20 (±2.02 SD) years, ranging from 18 to 30 years. Forty-seven (10.4%) were cigarette smokers. One hundred seven (23.6%) and 90 (19.9%) of the respondents drink alcohol and chewed khat, respectively. One hundred seventy-two (38%) of the respondents were exposed to pornographic materials at age < 18 years (Table 1).

### 3.2. Sexual History

Of the 453 respondents, 39.7% (180) of them ever had sexual intercourse. The mean age of sexual intercourse was 17.6 (±2 SD) years. Eighty-one (17.9%) had history of early sexual initiation. Nearly three-fourths (73.4%) of the respondent's reason for having sexual intercourse for the first time was falling in love. More than half (62.2%) of the respondents used a condom for their first sexual intercourse, and 82 (45.6%) had multiple sexual partner (Table 2).

### 3.3. Factors Associated with Early Sexual Initiation

Bivariate analysis was done for potentially expected associated factors. Independent variables found statistically significant at *p* < 0.25 in the bivariate analysis were included in the multivariable binary logistic regression model. Finally, sex, khat chewing, exposed to pornographic materials at age < 18 years, and knowledge of STDs were found to be significantly associated with early sexual initiation (Table 3).

## 4. Discussion

Early initiation of sexual activity affects the sexual and reproductive health of the young population [5, 13]. Based on the above scenario, this study is aimed at assessing the prevalence and associated factors of early sexual initiation among college students in southwest Ethiopia. As a result, the prevalence of early sexual initiation among college students was 17.9%, 95% CI (14.4%-24.4%). This study was in line with 21.9% in the 2016 Ethiopian Demographic and Health Survey [7], 20.4% in Ambo University [15], and 18.4% in Woldia town [5] studies done in Ethiopia. This study was higher than 7.1% in the United States of America [27]. This study was lower than 25.3% in Addis Ababa [9], 56.9% in Debre Markos University [25], and 60.6% in Dessie town and Dessie Zuria rural Woreda [6] studies in Ethiopia. The variation observed may be due to different factors. It could be due to the sociocultural and socioeconomic variation across different studies. Besides, the way to calculate the prevalence of early sexual initiation was varied across studies. Like that of this study, some studies used the participant population as denominator to calculate prevalence [5, 9], while some others used the number of study participants who had ever had sex as denominator [25, 32].

Female respondents were 2 times increased odds of having early sexual intercourse than male. Being female was significantly associated with early sexual initiation. This study was consistent with studies conducted elsewhere [6, 9, 25, 31]. This could be explained by the fact that female adolescents are more likely to compete with their peers with regard to have materials (fashion clothes, shoes, and latest electronic equipment), and this pushes them to have unwanted and early sexual intercourse with older rich individuals (what we call sugar daddy) to fulfill their needs. In contrary, adolescents engage in early, high-risk sexual activity and boys start at an earlier age than girls [1, 10, 11, 32].

Respondents who chewed khat were 7 times increased odds of having early sexual initiation than those who had not chewed khat. Chewing khat had a very strong association with early sexual initiation. This finding was consistent with previous studies done elsewhere [5–7]. Since individuals who chewed khat are more likely to drink alcohol, then the alcohol make their brain out of thinking what is good and what bad in that situation; this may lead to the unexpected initiation of early sexual intercourse with unconscious mind.

Respondents who exposed to pornographic materials at age < 18 years were 3.6 times increased odds of having early sexual intercourse than those who were not exposed. Being exposed to pornographic materials at age < 18 years was significantly associated with early initiation of sexual activity. This finding was consistent with other studies conducted in different areas [5, 6, 9, 32]. Adolescents who exposed to certain pornographic materials are more likely to think about the need to have sex again and again. This obsessive feeling and extreme need to have sex make adolescents prone for an inevitable and unwanted early sexual intercourse.

Having poor knowledge of STDs was strongly associated with early sexual initiation. Respondents who had poor knowledge of STDs were 8.7 times increased odds of initiating sexual activity as early as age 18 years. This study was supported by Nigussie et al. who revealed that good knowledge about STDs is strongly associated with majority of late sexual initiation [35]. This could be explained by the more you know about the disease symptoms, signs, and severity, the less chance of having sex at early age, which is a very risky sexual behavior that makes someone prone to various types of sexually transmitted diseases including HIV/AIDS.

## 5. Conclusion

The prevalence of early sexual initiation among college students was alarmingly high. This may be associated with a huge burden of poor sexual and reproductive health. Therefore, creating awareness of the factors and related negative sexual and reproductive health effect of early sexual initiation for young peoples through the use of mass media (television and radio), school teachers, and parents plays a paramount importance.

## Figures and Tables

**Table 1:** Sociodemographic and behavioral factors of the college students in southwest Ethiopia.

Variables	Categories	Frequency	Percent
Sex	Male	243	53.6
	Female	210	46.4
Age	<20 years	217	47.9
	≥20 years	236	52.1
Religion	Christian	392	86.5
	Muslim	61	13.5
Marital status	Out of marriage	407	89.8
	Within marriage	46	10.2
Residence	Rural	192	42.4
	Urban	261	57.6
Smoking cigarette	Yes	47	10.4
	No	406	89.6
Drinking alcohol	Yes	107	23.6
	No	346	76.4
Chewing khat	Yes	90	19.9
	No	363	80.1
Exposed to pornographic materials at age < 18 years	Yes	172	38
	No	281	62

**Table 2:** Sexual history of the college students in southwest Ethiopia.

Variables	Categories	Frequency	Percent
Sexually active (*n* = 453)	Yes	180	39.7
	No	273	60.3
Sexual initiation (*n* = 453)	Early	81	17.9
	Late	372	82.1
Relation of the first sexual partner (*n* = 180)	Boy/girlfriend	120	63.3
	Husband	24	17.7
	Unknown person	36	20
Reason for first sexual intercourse (*n* = 180)	Fall in love	132	73.4
	Got married	24	13.3
	Raped	5	2.8
	To get money	4	2.2
	Peer pressure	8	4.4
	Was drunk	7	3.9
Used condom for first sexual intercourse (*n* = 180)	Yes	112	62.2
	No	68	37.8
Number of sexual partner (*n* = 180)	<2	98	55.4
	≥2	82	45.6

**Table 3:** Factors associated with early sexual initiation of the college students in southwest Ethiopia.

Variables	Categories	Sexual initiation		COR (95% CI)	AOR (95% CI)	*p* value
		Early	Late			
Sex	Female	50	161	2.11 (1.29-3.46)^∗^	2.09 (1.17-3.75)	**0.013**
	Male	31	211	1	1	
Smoking cigarette	No	68	338	1	1	
	Yes	13	34	1.90 (0.95-3.79)	1.09 (0.48-2.46)	0.839
Drinking alcohol	Yes	26	91	1.46 (0.87-2.46)	0.84 (0.46-1.53)	0.571
	No	55	281	1	1	
Khat chewing	Yes	40	50	6.28 (3.71-10.7)^∗^	7.05 (3.81-13.1)	**<0.001**
	No	41	322			
Exposed to pornographic materials at age < 18 years	Yes	43	129	2.13 (1.31-2.3.47)^∗^	3.57 (1.94-6.59)	**<0.001**
	No	38	243	1	1	
Knowledge of STDs	Poor	75	201	10.6 (4.52-25.04)^∗^	8.69 (3.52-21.5)	**<0.001**
	Good	6	171	1	1	

CI: confidence interval; COR: crude odds ratio; AOR: adjusted odds ratio. ^∗^Significant at *p* value <0.01.

## Data Availability

The data set is handled by the corresponding author and can be provided upon request.

## Abbreviations

AOR: Adjusted odds ratio
CI: Confidence interval
COR: Crude odds ratio
SPSS: Statistical Package for the Social Sciences
SD: Standard deviation
STDs: Sexually transmitted diseases.
